# Nutritional, functional, and allergenic properties of silkworm pupae

**DOI:** 10.1002/fsn3.2428

**Published:** 2021-06-29

**Authors:** Xuli Wu, Kan He, Tanja Cirkovic Velickovic, Zhigang Liu

**Affiliations:** ^1^ Health Science Center School of Public Health Shenzhen University Shenzhen China; ^2^ Faculty of Chemistry Center of Excellence for Molecular Food Sciences and Department of Biochemistry University of Belgrade Belgrade Serbia; ^3^ Ghent University Global Campus Incheon South Korea; ^4^ Serbian Academy of Sciences and Art Belgrade Serbia

**Keywords:** allergen, allergy, food safety, function, insect protein, nutrition, silkworm pupae

## Abstract

Edible insects are a food source that has high nutritional value. Domestic silkworm pupae are an important by‐product of sericulture and have a long history as food and feed ingredients in East Asia. Silkworm pupae are a good source of protein, lipids, minerals, and vitamins and are considered a good source of nutrients for humans. Silkworm pupae are a valuable insect source of substances used in healthcare products, medicines, food additives, and animal feed. Because silkworm pupae are being increasingly used in the human diet, potential allergic reactions to the substances they contain must be elucidated. Here, we present an overview of the benefits of silkworm pupae. First, we describe their nutritional value. Second, we report their functional properties and applications, focusing on their potential use in the food and pharmaceutical industries. Finally, we consider the current state of research regarding silkworm pupae‐induced allergies.

## Abstract 2

Silkworm pupae have high nutritional value and varied biological activities. Silkworm pupae are a valuable insect source for healthcare products, medicine, food additives, and feed. Allergic reactions induced by silkworm pupae need attention and further research.

## 1 INTRODUCTION

Demand for sustainable sources of nutrition is increasing. Thus, edible insects, which have long formed part of the human diet in Asia, Africa, and Latin America (de Castro et al., 2018), have become a primary focus of scientific research in recent years. However, the allergic risks of consuming insects must be evaluated. Allergic reactions associated with eating edible insects range from a mild localized reaction to a more severe systemic clinical response. The main clinical symptoms include respiratory (e.g., dyspnea, asthma), gastrointestinal (e.g., nausea, vomiting, diarrhea), and skin (e.g., flushing, urticaria, rash, pruritus,) reactions (de Gier & Verhoeckx, 2018).

The silkworm (*Bombyx* *mori*) is a cultivated insect that is reared at large scales. Silkworm and its metabolites have high nutritional, medicinal, and economic value (Ratcliffe et al., 2011; Yang et al., 2009). The silkworm lifecycle encompasses four distinct developmental stages: ova, larva, pupa, and imago. Silk is obtained from the cocoon when silkworms transform from larvae to pupae.

The pupae of the two silk‐producing moths are edible. Silkworm pupae are frequently used as food in Asian countries, including Japan, Korea, India, and, especially, China (Zhou & Han, 2006). In China, silkworm pupae are eaten as food and are also an important component of traditional Chinese medicines used to treat hypertension and fatty liver (Zhang & Zhang, 2001). With increasing demand for sustainable animal‐derived dietary protein, edible insects are of interest as a source of protein (FAO, 2013). Silkworm pupae are considered a premium source of animal protein. They represent the only insect food in the List of Novel Food Resources published by the Ministry of Health of China and are widely used in dietary supplements, medicines, and animal feed in China and Korea (Kim et al., 2008; Zhu, 2004). In China, more than 100,000 tons of fresh silkworm pupae are produced annually (Dong & Wu, 2010). In recent years, silkworm pupae are used as raw materials in the food industry because of their high nutritional value and varied biological activities.

A major concern regarding novel sources of protein, including silkworm pupae, is their potential to cause food allergy (Pali‐Schöll et al., 2019). In recent years, large numbers of cases of allergic reactions to silkworm pupae have been reported (Ji et al., 2008), including serious allergies, and, even anaphylactic shock (van der Poel & Chen, 2009; Araujo et al., 2014). Thus, allergies to silkworm pupae are now the subject of extensive research worldwide.

This review describes the nutritional value of silkworm pupae, their functional properties, and potential applications. Within this framework, allergies to silkworm pupae are evaluated.

## 2 NUTRITIONAL VALUE OF SILKWORM PUPAE

Extensive data show that silkworm pupae have a well‐balanced nutrient profile, making them suitable as human food. Essentially, silkworm pupae are a good source of protein, fat, minerals, and vitamins. Compared to conventional food, they have higher content of three calorigenic nutrients (protein, fat, and carbohydrates), providing up to 230 kcal per 100 g. The values of key nutrients in silkworm pupae compared with other common foods are listed in Table 1.

**TABLE 1:** Key nutritional values per 100‐g silkworm pupae and other common foods

Name	Edible part (%)	Energy (kcal)	Water (g)	Protein (g)	Fat (g)	Carbohydrate (g)	VA (μg)	B1 (mg)	B2 (mg)	B3 (mg)	VE (mg)	Na (mg)	Ca (mg)	Fe (mg)
Silkworm pupae	100	230	57.5	21.5	13	6.70	0	0.07	2.23	2.2	9.89	140.2	81	2.6
Egg (white part)	87	138	75.8	12.7	9	1.50	310	0.09	0.31	0.2	1.23	94.7	48	2
Milk	100	54	89.8	3	3.2	3.40	24	0.03	0.14	0.1	0.21	37.2	104	0.3
Chicken	66	167	69	19.3	9.4	1.30	48	0.05	0.09	5.6	0.67	63.3	9	1.4
Pork (lean meat)	100	143	71	20.3	6.2	1.50	44	0.54	0.1	5.3	0.34	57.5	6	3
Sea shrimp	51	79	79.3	16.8	0.6	1.50	0	0.01	0.05	1.9	2.79	302.2	146	3
Tilapia	55	98	76	18.4	1.5	2.80	0	0.11	0.17	3.3	1.91	19.8	12	0.9

Note

Date sources: Food Ingredients Table of China (2017).

Abbreviations: B1, Thiamin; B2, Riboflavin; B3, Niacin; Ca, calcium; Fe, Iron; Na, Sodium; VA, Retinol; VE, Tocopherol.

The protein content of silkworm pupae is about 21.5%, which is higher than that of other typical animal products. On a dry‐weight basis, the protein content of silkworm pupae has been reported to be as high as 49%–54% (Longvah et al., 2011; Nowak et al., 2016). The proteins of silkworm pupae are considered complete proteins because of their high content of essential amino acids. In fact, silkworm contains all the amino acids required by the human body and in the appropriate proportions based on the recommendations of the FAO/World Health Organization (WHO) (Köhler et al., 2019; Ni et al., 2003; Wang et al., 2009; Yang et al., 2009; Zhou & Han, 2006). In general, insect proteins are highly digestible (Capinera and Finke, 2008). The amino acid score and protein digestibility‐corrected amino acid score of silkworm pupae are 100 and 86, respectively (Longvah et al., 2011). Furthermore, the amino acid score of proteins in silkworm pupae protein was 100 with respect to the amino acid profile of a 2–5‐year‐old child (FAO/WHO, 1985) (Longvah et al., 2011). Therefore, silkworm is a good source of protein that could be used as an alternative dietary source of protein for human nutrition.

Edible insects also have high fat content. In particular, dry silkworm pupae have a fat content of 25%–30% (Kouřimská and Adámková, 2016; Longvah et al., 2011). Silkworm pupae have high monounsaturated and polyunsaturated fatty acid contents and low saturated fat content (Payne et al., 2016). About 70%–80% of the fatty acid content of silkworm pupae is unsaturated, while about 1% is unsaponifiable matter in oil, including campesterol, β‐sitosterol, and cholesterol. In particular, silkworm pupae have almost 71% α‐linolenic acid content (Pereira et al., 2003; Rao, 1994; Zhao, Li, et al., 2015; Zhao, Wang, et al., 2015). Silkworm pupae are a good source of functional fatty acids. Furthermore, the oxidative stability of their total lipid content is very high, as the synergistic effect between tocopherol and phospholipids prevents the oxidation of lipids (Kotake‐Nara et al., 2002).

Some insect species, including weevil larvae and termites, have high quantities of saturated fat, however silkworm pupae do not (Payne et al., 2016). Based on WHO guidelines, limiting the dietary intake of saturated fat minimizes the risk of cardiovascular disease (World Health Organization (WHO), 2015). Silkworm pupae contain edible lipids of high quality that are used as raw materials in medicine (Shanker et al., 2006; Wang et al., 2013). For instance, after eating silkworm pupae oil for 18 weeks, rats showed a notable increase in high‐density lipoprotein cholesterol levels, with significantly reduced triglyceride and total cholesterol levels (Longvah et al., 2012). Silkworm pupae oil also regulates plasma lipid and lipoprotein levels in the serum of rats by activating apoproteins and lipid‐metabolizing enzymes. Thus, it could be used to treat hyperlipidemia (Hu & Chen, 2011). Therefore, silkworm pupae represent a good source of fats for human consumption.

Edible insects are also rich in minerals, including phosphorus, calcium, potassium, sodium, iron, zinc, magnesium, copper, and manganese (Kouřimská & Adámková, 2016). Silkworm pupae are a good source of calcium and iron (Table 1) and have high potassium content (34.0 mg/g), with a low Na/K ratio (0.08), and high zinc content (36 μg/g) (Zhou & Han, 2006), all of which are important for human health and nutrition. In addition, the heavy metal content (lead, arsenic, cadmium, and mercury) (Köhler et al., 2019) of silkworm pupae is below the maximum recommended level for human consumption and use in animal feed (National Standardization Management Committee of China, 2005). The mineral content reported in the published literature is highly varied (Köhler et al., 2019; Kouřimská & Adámková, 2016; Longvah et al., 2011; Nowak et al., 2016; Zhou & Han, 2006). These differences might be explained by mineral content varying with geographical location, seasonality, and diet, among other items. In general, edible insects contain a variety of vitamins, but have limited vitamin A (Kouřimská & Adámková, 2016). Silkworm pupae are a good source of thiamin, riboflavin, and niacin (Table 1). They contain about 10 mg tocopherols per 100 g, which is a much higher level than that found in other common foods. Importantly, the mineral and vitamin content of farm‐bred silkworm pupae can be controlled by regulating what they are fed.

Silkworm pupae also contain some antinutrients when they consume mulberry leaves. These antinutrients include phytate (72.89–110.16 mg/g) and phytin phosphorus (20.54–31.03 mg/g), as well as tannic acid, alkaloid, flavonoids, saponin, and oxalate. However, these antinutrients are present at low levels and fall within human tolerance levels. Thus, silkworm pupae are safe for human consumption (Omotoso, 2015).

Overall, silkworm pupae are a good source of protein, lipids, minerals, and vitamins, all of which are all important components of the human diet.

## 3 FUNCTIONAL PROPERTIES AND APPLICATIONS OF SILKWORM PUPAE

Silkworm pupae have many functional properties that could be utilized in the food and pharmaceutical industries. In recent years, various bioactive compounds have been found in silkworm pupae that have potential functions and health benefits. For instance, silkworm proteins and hydrolyzed peptides have several functions, including improving immunity, and antitumor and antioxidant activity (Table 2). Hydrolyzed peptides of silkworm pupae have multiple physiological functions. However, methods for their extraction, purification, and structural identification need to be developed, and the mechanisms of their pharmacological actions need describing. Current methods for preparing peptides from silkworm pupae by enzymatic hydrolysis generate poor yields. Thus, enzymatic hydrolysis must be optimized to facilitate the commercialization of this process. In addition, after deproteinization and degreasing, silkworm pupae residues contain 2%–8% chitosan, about 4% polysaccharides, and small amounts of antibacterial peptides and other bioactive materials, which have various biological activities (Battampara et al., 2020; Luo et al., 2010; Mishraa et al., 2003; Ni et al., 1998; Wang et al., 2007; Yue et al., 2013; Zhang et al., 2000). The precise functions of chitin and polysaccharides extracted from silkworm pupae in humans need further investigation. A dimethyladenosine compound extracted from silkworm pupae could be used as a vasorelaxant to treat vasculogenic impotence in men (Ahn et al., 2008). Many natural drugs have been isolated from microbes, marine organisms, and plants; however, few drugs isolated from insects are available on the market. The silkworm has long been used in Chinese medicine (Ratcliffe et al., 2011). Scientific studies have demonstrated the health benefits of silkworm pupae, and it has been reported that eating silkworm pupae does not cause mutation or hepatotoxicity (Yang et al., 2010; Zhou & Han, 2006). Thus, silkworm pupae represent a valuable insect source for use in medicines and other healthcare products. Although some active substances in silkworm pupae have been identified, their pharmacological actions remain unclarified. With in‐depth research, silkworm pupae have great potential for use in the pharmaceutical industry.

**TABLE 2:** Functions of proteins and hydrolysis peptides in silkworm pupae

Source	Function	Studies conducted
Protein Hydrolysis peptide	Antifatigue Antifatigue	(Xu & Lu, 2008; Zhang et al., 2005) (Chen et al., 2009; Wen et al., 2009)
Hydrolysis peptide	Antihypertensive property	(Wei et al., 2008; Wang et al., 2011; Wu et al., 2011; Zhao et al., 2011; Zhang et al., 2005; Wu et al., 2015; Wang et al., 2014)
Extract Hydrolysis peptide	Antioxidant activity Antioxidant activity	(Deori et al., 2014) (Li et al., 2011; Wang et al., 2012; Min et al., 2009; Xiao et al., 2011; Lu et al., 2013; Zhao et al., 2011; Zhang et al., 2021)
Protein Hydrolysis peptide	Enhance/regulate immune responses Enhance/regulate immune responses	(Yeo et al., 2013) (Li et al., 2019; Liu et al., 2010; Lu et al., 2013; Qi et al., 2009; Yang et al., 2008)
Hydrolytic amino acid	Antitumor activity	(Hu et al., 2004; Li et al., 2018; Yan et al., 2008; Zhan et al., 2013, 2015)
Extract Hydrolysis peptide	Weight reduction Weight reduction	(Ryu, 2014) (Lee et al., 2012)
Extract	Alcohol detoxification	(Kwon et al., 2012)
Hydrolysis peptide Hydrolysis peptide	Serum glucose reduction Decrease fat accumulation	(Zhang et al., 2016; Lee et al., 2011) (Lee et al., 2011)
Hydrolysis peptide	Umami taste enhancement	(Yang et al., 2017)

A study on the functional properties of silkworm pupae demonstrated their potential for use as food additives (Omotoso, 2015). In addition to direct consumption, the protein powder of silkworm pupae has been used as a food additive in cookies, noodles, bread, and other foods. Kim et al. (2016) used flour from silkworm pupae to replace 10% lean pork as a novel protein ingredient and showed that it enhanced the hardiness and cooking yield of emulsion sausages.

One of the greatest barriers to the adoption of edible insects as a direct food is consumer acceptance (Mlcek et al., 2014). Although consumers recognize the high nutrition value of insects, they prefer to not know what they are eating in this case (Govorushko, 2019). Certain components extracted from insects are preferable for consumers. Thus, extracting the proteins of silkworm pupae, or other bioactive compounds, could increase consumer acceptability. Future research should focus on developing methods to extract and process the proteins of silkworm pupae for use in the food industry.

Compound amino acids prepared by the hydrolysis of silkworm pupae could be easily accepted by consumers. In recent years, degreased silkworm pupae have been used in China to mass‐produce compound amino acids for use in health and medical care. A total of 11 healthcare products using compound amino acids derived from the proteins of silkworm pupae have been approved by the Ministry of Health and the State Food and Drug Administration in China and are commercially available on the Chinese market. Eight of these products enhance immune response, while two products are used to combat fatigue (http://www.samr.gov.cn/fw/wyc/).

Insects successfully substitute many ingredients used in the commercial production of animal feed (Govorushko, 2019). Silkworm pupae are used as fishmeal for breeding poultry, fish, and crustacean species (Konwar et al., 2008; Rahimnejad et al., 2019; Sun et al., 2014). This use of silkworm pupae could help to reduce other sources of protein feedstuffs, with positive effects on molting time, antioxidant capacity, and digestibility. Besides consuming silkworm directly or as ingredients in food, the human need for food can be met by using silkworm pupae as an animal feedstuff.

In recent years, the number of patents for inventions associated with silkworm pupae has increased rapidly in China. According to patent data from the State Intellectual Property Office of China, between 1989 and October 2019, there were 476 invention patents associated with silkworm pupae, of which 257 were issued between 2014 and October 2019. Of these 476 patents, 276 were associated with human needs. Of these 246 patents, 86 patents were for human medicine, veterinary medicine, forestry, agriculture, hunting, animal husbandry, fishing, trapping, and baking. The other 189 patents were for use in foods or food ingredients and their processing (not covered by other categories). Thus, patent applications for silkworm pupae as food and animal feed are rapidly increasing in China. Many patents for silkworm pupae have also been issued in South Korea, with applications including snack manufacturing and the refining of silkworm powder (Han et al., 2017).

## 4 SILKWORM ALLERGY

### 4.1 Silkworm‐induced allergy

The possible allergic reactions associated with eating insects are of concern for both consumers and scientists. Safety is a vital factor in food quality, with it being important to build confidence in the consumption of edible insects. Silkworms contain important sources of allergens, including excrement, dander, and silk. These allergens can cause various allergic reactions, such as cutaneous symptoms, respiratory symptoms, cardiovascular symptoms, and, even, anaphylactic shock (Gautreau et al., 2017; Ji et al., 2008; Vovolis & Galatas, 1999). These reactions could limit the use of silkworm pupae in food and feed. Potential silkworm‐induced allergies include respiratory allergy, contact allergy, and food allergy.

The silkworm is widely cultivated, and its silk is widely used. During processing and use, silk particles and wild silk floating in the air might be inhaled, causing allergies (Häcki et al., 1982; Inoue et al., 1997). Furthermore, inhaling the scales of the silkworm moth suspended in the air might cause respiratory allergies (Blanc et al., 1999; Suzuki et al., 1995). People allergic to silkworm might have a reaction when they are in contact with silk products (contact allergy) (Borelli et al., 1999; Ohansson et al., 1985). Silkworm pupae also cause allergic reactions when consumed as food (Ji et al., 2008; Choi et al., 2010). Thus, allergens likely exist at different developmental stages of the silkworm lifecycle; however, it is not clear whether these allergens are the same protein.

Silkworm pupae are consumed raw, boiled, and fried in several Asian countries. Allergic reactions after consuming silkworm pupae include urticaria, dizziness, skin itching, and shock (Feng et al., 2018). In China, many cases of silkworm pupae‐induced allergies have been reported. Each year, at least 1,000 patients have anaphylactic reactions after eating silkworm (Ji et al., 2008). In Korea, 9.4% of patients were found to be allergic to silkworm pupae (Kim et al., 2003). In East Asia, allergic reactions to eating silkworm pupae are considered a common cause of anaphylaxis (van der Poel & Chen, 2009). However, allergic reactions caused by silkworm pupae are no more serious than those caused by conventional foods, such as shrimp and fish (de Gier & Verhoeckx, 2018). Because silkworm pupae are being increasingly incorporated into the daily human diet, the mechanisms driving these allergic reactions need to be elucidated.

### 4.2 Allergens present in silkworm pupae

To date, 26 silkworm pupae proteins have been identified as allergens (Table 3). The only protein characterized as an allergen in silkworm larvae was an arginine kinase, which cross‐reacted with an arginine kinase in cockroaches (Liu, Wu, et al., 2009; Liu, Xia, et al., 2009). However, except for this arginine kinase (officially named Bomb m 1) (Liu, Wu, et al., 2009; Liu, Xia, et al., 2009), no allergens of silkworm pupae have been officially confirmed and registered by the WHO and International Union of Immunological Societies (WHO/IUIS) Allergen Nomenclature Sub‐committee (www.allergen.org). Five allergen proteins belong to lipoprotein 11 family (30‐kDa family proteins), while three are insect cuticle proteins (Table 3). The other proteins belong to different protein families. According to the WHO/IUIS Allergen Nomenclature Sub‐committee, proteins reported as allergens in other species include heat shock protein, paramyosin, chitinase, profilin, triosephosphate isomerase, and vitellogenin.

**TABLE 3:** Allergens in silkworm pupae

Allergen	Molecular weight (KDa/PI)	Identification method	GI No	Family	Reference
Juvenile hormone epoxy hydrolase	52.36/6.08	WB	255977192	Abhydrolase 1 superfamily	(Jeong et al., 2016)
Cell differentiation protein	32.17/7.06	WB	290760296	Cell differentiation family	(Jeong et al., 2016)
Chitinase precursor	61.52/5.78	WB	29467722	Chitinase A *N*	(Zhao et al., 2015)
27‐kDa glycoprotein	24.89/5.12	WB/ELISA	19911074	DUF1397	(Jeong et al., 2016)
Cuticular protein RR−2 motif 63 precursor	18.5/6.61	WB	290563229	Insect cuticle protein	(Liang et al., 2016)
Putative cuticle protein	45.68/4.14	WB	223671172	Insect cuticle protein	(Jeong et al., 2016)
Putative cuticle protein	28,28/4.63	WB	19911074	Insect cuticle protein	(Jeong et al., 2016)
Chemosensory protein 5 precursor	14.26/6.89	WB/ELISA	112983054	Insect pheromone‐binding family	(Ma et al., 2016)
Cellular retinoic acid binding protein	14.86/5.66	WB	112983600	Lipocalin/cytosolic fatty acid binding protein	(Liang et al., 2016)
30‐kDa protein precursor	29.39/unknown	WB/Murine model of asthma	112984502	Lipoprotein 11	(Zuo et al., 2015)
30‐kDa lipoprotein precursor	30/unknown	WB	154201552	Lipoprotein 11	(Cao et al., 2017)
Mature 30‐kDa lipoprotein	28.55/6.37	WB	1335608	Lipoprotein 11	(Zuo et al., 2015)
LOC114242470 low molecular mass 30‐kDa lipoprotein 21G1	30.34/6.33	WB	266439	Lipoprotein 11	(Zuo et al., 2015)
Low molecular mass 30‐kDa lipoprotein 19 G1 precursor	29.65/6.9	WB	266438	Lipoprotein 11	(Zuo et al., 2015)
Heat shock protein 20.8	20.8/unknown	WB/ELISA	148298693	Metazoan ACD (Alpha‐crystallin domain of metazoan)	(Zuo et al., 2015)
Paramyosin	10.28/5.43	WB	195963325	Myosin tail 1	(Zhao et al., 2015)
Thiol peroxiredoxin	22/unknown	WB/ELISA/Murine model of asthma	112982996	Peroxiredoxin (PRX) family	(Wang et al., 2016)
AGAP008849‐PA	35.88/8.09	WB	58392734	Phosphoglycerate dehydrogenases	(Zuo et al., 2015)
Xanthine dehydrogenase	147.63/6.38	WB	13936381	PLN02906 superfamily	(Jeong et al., 2016)
Profilin	14/unknown	WB	118500453	Profilin	(Hu et al., 2017)
Triosephosphate isomerase	26.93/5.67	WB	125987,66	PTZ00333	(Zuo et al., 2015)
Chymotrypsin inhibitor CI‐8A	43.91/5.2	WB	14028769	Serpin	(Zuo et al., 2015)
Tropomyosin	40/unknown	WB	195963325	Tropomyosin	(Liu et al., 2009)
Uncharacterized protein LOC101743840	33/6.76	WB	1200729467	unknown	(Liang et al., 2017)
Cuticular protein hypothetical 30	25/unknown	WB	290560816	unknown	(Hu et al., 2016)
Vitellogenin	20.35/7.07	WB	32526658	Vitellogenin *N*	(Zuo et al., 2015)

Abbreviation: WB, Western blot analysis.

Heat shock protein, tropomyosin, chitinase, and triosephosphate isomerase are also allergens of house dust mites; their official names are Der f 10, Der f 11, Der f 15, and Der f 25, respectively. The National Center for Biotechnology Information (www.ncbi.nlm.nih.gov) showed that the sequence homologies of amino acids in silkworm pupae and house dust mites are 30.77%, 81.94%, 28.33%, and 71.77% for heat shock protein, tropomyosin, chitinase, and triosephosphate isomerase, respectively. Tropomyosin is a well‐known invertebrate pan‐allergen and is a major allergen of crustaceans, house dust mites, cockroaches, and moths (Ivanciuc et al., 2009). Arginine kinase is another well‐known invertebrate pan‐allergen. It is an enzyme found in insects and crustaceans, belonging to the guanidino phosphotransferase family (Azzi et al., 2004). However, arginine kinase has been reported as an allergen of silkworm larvae, but not of silkworm pupae. Wang and Xu (2006) showed that the gene for arginine kinase is also expressed in pupae at a relatively low level, with expression increasing as larvae mature. Thus, studies are needed to examine the allergenicity of arginine kinase in silkworm pupae. Vitellogenin is an allergen of the honey bee (Api m 12), chum salmon (Onc k 5), and yellow jacket (Ves v 6). Profilin is a pan‐allergen (McKenna et al., 2016). The homology of arginine kinases and tropomyosins from different insect species is greater than 70%, while that of other allergenic proteins ranges from 35% to 95%. This phenomenon might induce cross‐reactive reactions between proteins from different insect species and from crustaceans, such as shrimp (de Gier & Verhoeckx, 2018). Thus, the allergens of some silkworm pupae have high homology and structural similarity with those from other species, with cross‐reactive potential. Cross reactions between invertebrate proteins are usually attributed to homologous allergens (Marti et al., 2007). Silkworms belong to the phylum Arthropoda, which includes crustaceans. A comparative proteomic study showed that some pupae proteins are differentially expressed when comparing gender and diet of silkworm larvae (Lamberti et al., 2019). Of note, the levels of known allergens in female silkworm pupae reared on mulberry leaves were lower compared to those reared under other experimental conditions (Lamberti et al., 2019). Thus, the allergen profile of silkworm pupae could be associated with gender and diet and so could be regulated.

In recent years, there has been extensive research to minimize food allergenicity in technical food processing. However, only the 27‐kDa hemolymph glycoprotein has been identified as a heat‐stable allergen in silkworm pupae (Jeong et al., 2016). Knowledge remains limited on how food processing affects the allergenic potential of proteins in silkworm pupae. Methods to minimize allergens in silkworm pupae need to be developed. In the food industry, only hydrolysis and fermentation have achieved a satisfactory reduction in food allergenicity (Lepski & Brockmeyer, 2013; Verhoeckx et al., 2015). These processes might have similar effects on silkworm pupae. Enzymatic hydrolysis represents a promising processing technology for exploiting the bioactive properties of silkworm pupae. This process might also represent an effective means of minimizing allergenicity. Through understanding the structure–allergenicity relationship of allergens in silkworm pupae, effective methods to process hypoallergenic proteins could be developed. Further studies are required to characterize the immunological properties of allergens in silkworm pupae.

## 5 PERSPECTIVES AND CONCLUSIONS

The silkworm is an insect that can be produced at a large scale. After extracting the silk thread, silkworm pupae are a subproduct of the textile industry. These pupae are rich in nutrients and have high value. The potential uses of silkworm pupae have attracted increasing research interest. Many studies have shown that silkworm pupae are a good source of nutrients for humans, with various potential functions. Although silkworm pupae have many benefits for humans, consumer acceptance remains low, especially in Western countries. Good cooking methods could promote the consumption of insects as food (Feng et al., 2018). Furthermore, the incorporation of insects into everyday food items is a documented strategy that overcomes the challenges associated with the low acceptance of insects as food (Khatun et al., 2021; Bawa et al., 2020; Nyangena et al., 2020). For instance, consuming protein powder and hydrolyzed peptides in a food product might be more acceptable than direct consumption.

However, developing silkworm pupae as a food source requires an effective risk assessment of potential allergenic hazards. Evaluation of the allergies of silkworm pupae is scarce and is primarily based on case reports without the consistent use of standardized tests. Overall, 26 silkworm pupae proteins have been identified as allergens; however, their immunological properties remain poorly characterized.

Potential strong cross reactions of silkworm pupae allergens should be carefully examined. Large‐scale investigations of such cross‐reactions are required. The detection, labeling, and elimination of silkworm pupae allergens is needed to combat allergic reactions. Thus, the allergens present in silkworm pupae, and the mechanisms by which they cause allergic reactions, must be fully elucidated.

## CONFLICT OF INTEREST

The authors declared they do not have any conflict of interest.

## AUTHOR CONTRIBUTIONS

**Xuli Wu:** Conceptualization (lead); Funding acquisition (lead); Methodology (lead); Project administration (lead); Resources (lead); Supervision (lead); Validation (lead); Visualization (lead); Writing‐original draft (equal); Writing‐review & editing (lead). **Kan He:** Investigation (lead); Writing‐original draft (equal). **Tanja Cirkovic Velickovic:** Conceptualization (equal); Writing‐original draft (equal). **Zhigang Liu:** Resources (equal); Writing‐original draft (equal).

## ETHICAL APPROVAL

This study does not involve any human or animal testing.

## Data Availability

The data that support the findings of this study are available on request from the corresponding author.
